# Four species of spider genus *Cheiracanthium* C. L. Koch, 1839 (Araneae, Eutichuridae) from Jinggang Mountains, Jiangxi Province, China

**DOI:** 10.3897/zookeys.762.23786

**Published:** 2018-05-30

**Authors:** Jianshuang Zhang, Guren Zhang, Hao Yu

**Affiliations:** 1 School of Life Sciences, Guizhou Normal University, Guiyang, Guizhou, China; 2 State Key Laboratory for Biocontrol, Sun Yat-Sen University,; 3 Guangzhou, Guangdong, China; 4 College of Chemistry and Life Sciences, Integrated Mountain Research Institute, Guizhou Education University, Guiyang, Guizhou, China

**Keywords:** Taxonomy, morphology, description, new species

## Abstract

Four species of spider genus *Cheiracanthium* C. L. Koch, 1839 are reported from Jinggang Mountains, Jiangxi Province, China. Two of them are described as new to science: *C. auriculatum* **sp. n.** (♀♂) and *C. echinulatum* **sp. n.** (♂). *Cheiracanthium taiwanicum* Chen, Huang, Chen & Wang, 2006 is recorded from Mainland China for the first time. *Cheiracanthium zhejiangense* Hu & Song, 1982, the most similar species to *C. auriculatum* **sp. n**., is a newly recorded species of Jiangxi Province. Detailed descriptions, diagnoses, and photographs of the two new species are given. *Cheiracanthium taiwanicum* and *C. zhejiangense* are also illustrated.

## Introduction

*Cheiracanthium* C. L. Koch, 1839 contains 210 catalogued species and is mainly distributed in the Old World (Marusik and Fomichev 2016; World Spider Catalogue 2018). Although this genus is relatively large and well known, its taxonomy is rather poorly studied. Almost half of its species are known from single sex or juveniles: 36 by males, 60 by females, 2 by juveniles (World Spider Catalogue 2018). Additionally, 16 species were never illustrated and many species were described based on poor illustrations. So far, the genus has not been the subject of any global or regional revisions (Marusik and Fomichev, 2016).

The *Cheiracanthium* fauna of China is relatively rare and poorly represented, with only 38 described species (Li and Lin 2016; World Spider Catalogue 2018), of which 14 species are known based on a single sex: for 11, only females are known, and for three, only males are known (World Spider Catalogue 2018). Additionally, illustrations of the internal structure of the epigyne are not provided in five species (World Spider Catalogue 2018). Moreover, the diversity of this genus in China is still insufficiently known and several new species have been described in the last few years (Chen and Huang 2012; Barrion et al. 2013; Wang and Zhang 2013).

Field collection in Jinggang Mountains of Jiangxi province, China, was carried out in April 2011. During this field exploration, four *Cheiracanthium* species were found: *C. auriculatum* sp. n., *C. echinulatum* sp. n., *C. taiwanicum* Chen, Huang, Chen & Wang, 2006 and *C. zhejiangense* Hu & Song, 1982. Descriptions and photographs of the new species, as well as supplementary micrographs of the known species, are provided.

## Materials and methods

Spiders were fixed and preserved in 80% ethanol. Specimens were examined with an Olympus SZX7 stereomicroscope; details were studied with an Olympus BX51 compound microscope. Male palps and female epigynes were examined and illustrated after being dissected. Epigynes were cleared in boiling KOH solution to dissolve soft tissues. Photographs were made with a Leica DFC450 digital camera mounted on an Olympus BX51 compound microscope. The digital images were taken and assembled using Helicon Focus 3.10 software package.

All measurements were obtained using an Olympus SZX7 stereomicroscope and given in millimetres. Eye diameters are taken at the widest point. The total body length does not include chelicerae or spinnerets length. Leg lengths are given as total length (femur, patella, tibia, metatarsus, tarsus). The type specimens of the new species are deposited in College of Chemistry and Life Sciences, Guizhou Education University, Guiyang, Guizhou, China.

**Abbreviations used are**:

**A** atrium;

**AER** anterior eye row;

**AL** abdomen length

**ALE** anterior lateral eyes;

**AME** anterior median eyes;

**AME–AME** distance between AMEs;

**AME–ALE** distance between AME and ALE;

**AW** abdomen width;

**C** conductor;

**CD** copulatory duct;

**CF** cymbial fold;

**CI** carapace index;

**CL** carapace length;

**CLL** clypeal length;

**CO** copulatory opening;

**CS** cymbial spur;

**CW** carapace width;

**DTA** dorsal tibial apophysis;

**E** embolus;

**EB** embolic base;

**FD** fertilisation duct;

**LL** total length of leg I;

**LL:CL** leg I / carapace length;

**MOQ** median ocular quadrangle;

**MOQA** MOQ anterior width;

**MOQP** MOQ posterior width;

**OAL** ocular area length;

**OAW** ocular area width;

**PER** posterior eye row;

**PLE** posterior lateral eyes;

**PME** posterior median eyes;

**PME–PME** distance between PMEs;

**PME–PLE** distance between PME and PLE;

**PTA** prolateral tibial apophysis;

**RTA** retrolateral tibial apophysis;

**R** receptacle;

**STL** sternum length;

**STW** sternum width;

**TA** tegular apophysis;

**TL** total body length.

Leg setae: v, ventral; p, prolateral; r, retrolateral. Most of the terminologies used in text and figure legends followed Lotz (2015), while a few others followed Marusik and Fomichev (2016) and Morano and Bonal (2016).

## Taxonomy

### Genus *Cheiracanthium* C. L. Koch, 1839

#### Cheiracanthium auriculatum sp. n.

Taxon classificationAnimaliaAraneaeEutichuridae

http://zoobank.org/45045AEA-20E9-4C6E-8BBD-F104775A8E6B

Figs 1 , 5

##### Type material.

Holotype ♂ (SYSU-JX-11-177): China, Jiangxi Province, Jinggang Mountains Nature Reserve, Xiangzhou village (380 m; 26°35'30.23"N, 114°15'59.93"E), 26 April 2011, Hao Yu and Zhenyu Jin leg. Paratypes: 1♂ and 3 ♀, same data as holotype.

##### Etymology.

The specific epithet is an adjective and is derived from a Latin word “*auriculatus*“(ear-like), referring to the tegular apophysis which is like the contour of an ear in ventral view.

##### Diagnosis.

**Figures 1.**
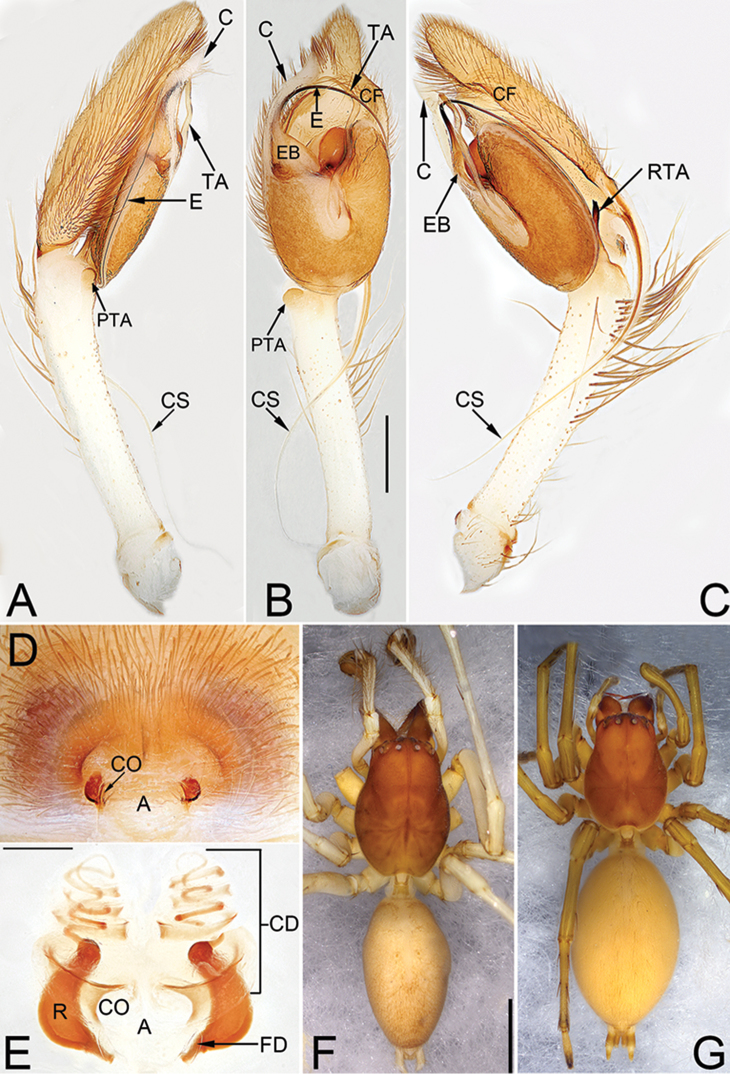
*Cheiracanthium auriculatum* sp. n., male holotype and female allotype. **A** left palp, prolateral view **B** same, ventral view **C** same, retrolateral view **D** epigyne, ventral view **E** vulva, dorsal view **F** male habitus, dorsal view **G** female habitus, dorsal view. Scale bars: 0.5 mm (**A–C**); 0.2 mm (**D–E**); 2 mm (**F–G**).

*Cheiracanthium auriculatum* sp. n. is distinguished from all other *Cheiracanthium* species, except *C. zhejiangense* Hu & Song, 1982 (Fig. 4A–E), by having a distally filiform cymbial spur in the male, and by the general shape of the vulva in the female. From *C. zhejiangense*, the male can be distinguished by the ear contour-shaped tegular apophysis and the uncoiling tip of cymbial spur (*vs* the falciform tegular apophysis and the coiled tip of the cymbial spur in *C. zhejiangense*) (Figs 1A–C; 4A–C), the female can be differentiated by the indistinct atrium and copulatory ducts (*vs* the distinct atrium and copulatory ducts in *C. zhejiangense*), the more or less lengthwise receptacles (*vs* the nearly horizontal receptacles in *C. zhejiangense*) (Figs 1D–E; 4D–E), and by the different coil number of copulatory ducts (7 coils in *C. auriculatum* sp. n., instead of 8 coils in *C. zhejiangense*) (Figs 1E; 4E). In addition, the two species can by separated by their habitus: abdomen without distinct colour pattern in *C. auriculatum* sp. n. (Fig. 1F–G), but with a median heart-shaped mark which reaches half of the opisthosoma length in *C. zhejiangense* (Fig. 4F–G).

##### Description.

***Male***. Total length 8.58–9.15. Holotype (Fig. 1A–C, F): TL 9.15; CL 3.73, CW 2.41, CI (CL/CW) 1.55; AL 4.05, AW 2.42. *Carapace* (Fig. 1F) brown, uniformly coloured, without distinct pattern. Eye sizes and inter-distances: OAL 0.39, OAW 1.45; AME 0.14, ALE 0.16, PME 0.16, PLE 0.17; AME–AME 0.27, AME–ALE 0.27, PME–PME 0.36, PME–PLE 0.35; MOQA 0.56, MOQP 0.67, CLL 0.10. *Chelicerae* protruding and reddish brown, with 3 teeth on promargin and 3 on retromargin respectively. *Sternum* dark brown, STL 1.69, STW 1.44. Labium and endites brown. *Legs* yellowish-white, without distinct colour markings. Leg measurements: I 18.84 (4.65, 1.22, 5.10, 5.60, 2.26), II 12.09 (3.30, 1.14, 3.24, 3.23, 1.19), III 8.61 (2.38, 0.86, 1.92, 2.40, 1.05), IV 12.47 (3.72, 0.95, 3.17, 4.00, 1.07); LL:CL 5.03. Leg spines: I 0-0-1p, 2v-1v-1p, 2v1p-1p1v-1v; II 0-0-1p, 1v-2v-1p, 2v1p-1v1p-1v; III 0-0-1p1r, 0-1p1r-0, 2v1p-1p1r-1v2p2r; IV 0-0-1p1r, 1v-1v1p-0, 2v1p-1v1p1r-1v2p2r. *Abdomen* (Fig. 1F) elongate-oval, dorsally grey, dorsum with indistinct heart-shaped mark and two pairs of not obvious muscle depressions; venter brownish without distinct pattern.

*Palp* (Fig. 1A–C). Tibia extremely long, about as long as cymbium, with two apophyses; retrolateral tibial apophysis (RTA) about 20% of tibia length, with a more or less bifurcate apex and hiding behind tegulum; prolateral tibial apophysis (PTA) small and round; cymbial spur (CS) is approximately equal in length to tibia, tapering off into a filiform; cymbial fold (CF) poorly developed, for approximately 4/5 the length of cymbium; tip of cymbium short, about 1/4 of cymbium length. Tegulum oblong, 1.3 times longer than wide; tegular apophysis (TA) long and sinuate, more than 4/5 of tegulum length, filamentous and like an ear’s contour in ventral view; embolus (E) arising at approximately 10 o’clock position, terminating at approximately 11 o’clock position, it’s tip covered by conductor (C); conductor large, falciform.

***Female***. Total length 8.66–9.30. Slightly larger in size and lighter in colour. Allotype (Fig. 1D–E, G) measured: TL 9.30; CL = 3.03, CW = 2.22, CI (CL/CW) = 1.36; AL = 4.95, AW = 2.92. Eye diameters and inter-distances: OAL 0.37, OAW 1.23; AME 0.14, ALE 0.19, PME 0.13, PLE 0.14; AME–AME 0.23, AME–ALE 0.11, PME–PME 0.31, PME–PLE 0.23; MOQA 0.46, MOQP 0.58, CLL 0.24. PMT: RMT = 6:6.STL 1.47, STW 1.23. Leg measurements: I 12.70 (3.30, 1.06, 3.42, 3.39, 1.54), II 8.51 (2.42, 0.86, 2.23, 2.05, 0.96), III 6.42 (1.92, 0.69, 1.35, 1.65, 0.80), IV 9.67 (2.75, 0.89, 2.39, 2.68, 0.96); LL:CL 4.19. Leg spines: I 0-1p-1p, 2v-2v-0, 2v-1p1r-1v; II 0-0-1p, 1v-2v-1p, 2v1p-1p1r-1v; III 0-1p-1p1r, 1v-1p1r-0, 2v1p1r-1p1r-1v2p2r; IV 0-0-1p1r, 1v-1v1p1r-0, 2v1p1r-1v1p1r-1v2p2r.

*Epigyne* (Fig. 1D–E). Atrium (A) indistinct, without delimited margin, about four times wider than long; receptacles (R) are faintly visible through epigynal plate in ventral view; two copulatory openings (CO) located at lateral borders of atrium; the transparent copulatory ducts (CD) running spirally (length of spira about 1.4 times longer than receptacles), forming 7 entwined loops (including 4 ascending coils and 3 descending coils); receptacle sickle-shaped, separated by three diameters.

##### Distribution.

Presently known only from the type locality, Jinggang Mountains, Jiangxi, China (Fig. 5).

#### Cheiracanthium echinulatum sp. n.

Taxon classificationAnimaliaAraneaeEutichuridae

http://zoobank.org/A1935AC9-A0A9-45F2-8BFA-22E0F3172401

Figs 2 , 5

##### Type material.

Holotype ♂ (SYSU-JX-11-182): China, Jiangxi Province, Jinggang Mountains Nature Reserve, Xiangzhou village (380 m; 26°35'30.23"N, 114°15'59.93"E), 26 April 2011, Hao Yu and Zhenyu Jin leg. Paratypes: 1♂, same data as holotype.

##### Etymology.

The species epithet is taken from the Latin adjective *echinulatus* and refers to the spinule-shaped tegular apophysis.

##### Diagnosis.

**Figures 2.**
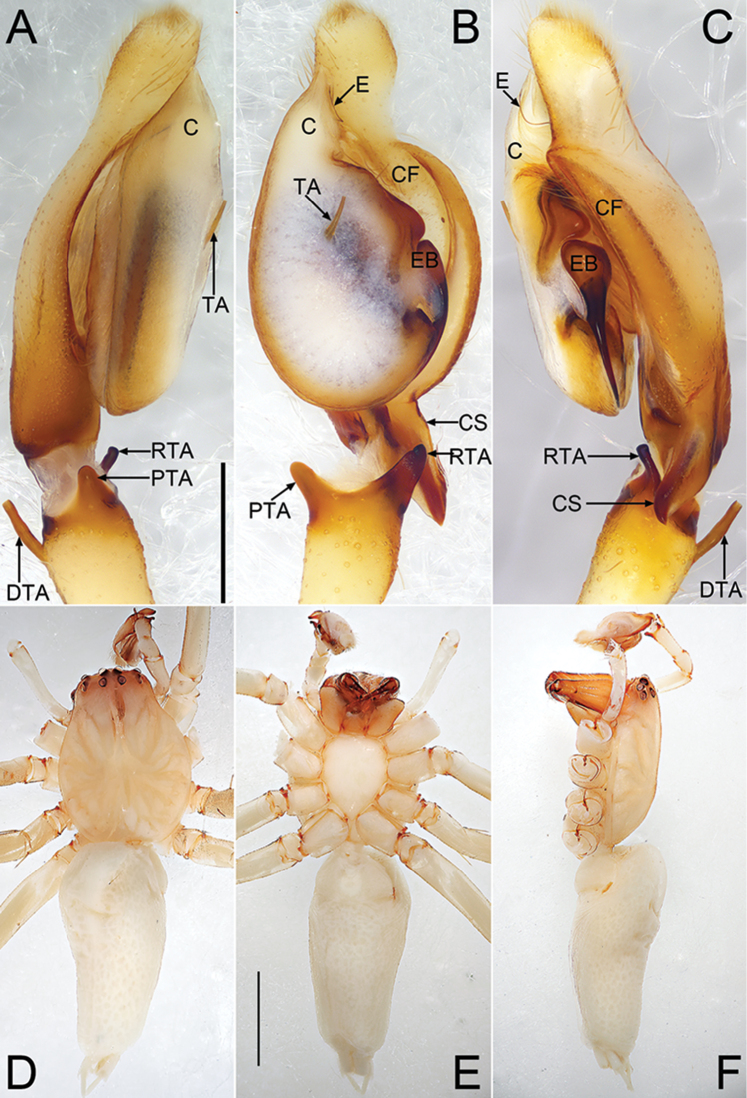
*Cheiracanthium echinulatum* sp. n., male holotype. **A** left palp, prolateral view **B** same, ventral view **C** same, retrolateral view **D** male habitus, dorsal view **E** same, ventral view **F** same, lateral view. Scale bars: 0.5 mm (**A–C**); 2 mm (**D–F**).

This new species resembles *C. taegense* Paik, 1990 (Paik, 1990: 11, f. 39–47; Baba & Yoshitake, 2016: 39, f. 1–4) in having the similar beak-shaped cymbial spur, and stalk-like dorsal tibial apophysis, but can be distinguished by: (1) the embolus originated at 3 o’clock position (Fig. 2B–C), *vs.* originated at 1–2 o’clock position in *C. taegense* (Paik, 1990: 11, f. 41; Baba & Yoshitake, 2016: 39, f. 3–4); (2) tegular apophysis smaller, straight and acicular (Fig. 2A–C), instead of bigger and slightly curved in *C. taegense* (Paik, 1990: 11, f. 41, 43; Baba & Yoshitake, 2016: 39, f. 3–4); (3) RTA straight and digitiform (Fig. 2A–C), but with a curved and hook-shaped apex in *C. taegense* (Baba & Yoshitake, 2016: 39, f. 3–4).

##### Description.

***Male***. Total length 9.06–9.12. Holotype (Fig. 2): TL 9.06; CL 3.58, CW 2.34, CI (CL/CW) 1.53; AL 4.99, AW 2.27. *Carapace* (Fig. 2D, F) yellow except reddish ocular area, without distinct colour pattern. Eye sizes and inter-distances: OAL 0.34, OAW 1.26; AME 0.15, ALE 0.15, PME 0.14, PLE 0.13; AME–AME 0.46, AME–ALE 0.25, PME–PME 0.54, PME–PLE 0.22; MOQA 0.43, MOQP 0.55, CLL 0.13. *Chelicerae* light brown and robust, with long and wine-coloured fangs, with 3 teeth on promargin and 3 on retromargin respectively. *Sternum* (Fig. 2E) yellowish, STL 1.79, STW 1.32. Labium and endites brown. *Legs* yellowish, without distinct colour markings. Leg measurements: I 23.70 (5.90, 1.05, 7.31, 7.99, 1.46), II 14.92 (3.41, 0.82, 4.14, 5.26, 1.29), III 11.10 (2.53, 1.43, 2.16, 3.94, 1.03), IV 16.42 (4.18, 1.21, 4.13, 5.66, 1.24); LL:CL 6.62. Leg spines: I 0-1p1r-1p1r, 3v-3v-1v1p, 2v-0-1v; II 0-1p1r-1p1r, 3v-2v-1v1p, 2v1p-2v1p-1v; III 0-1p1r-1p1r, 2v1p1r-1p1r-0, 2v1p1r-2v1p1r-2v1p2r; IV 0-1p1r-1p1r, 1v1p1r-1v2r-1v1r, 2v1p1r-2v1p1r-1v1p3r. *Abdomen* (Fig. 2D–F) lanceolate, dorsally yellowish white, scattered numerous indistinct pigmented spots; venter yellowish without distinct pattern.

*Palp* (Fig. 2A–C). Tibia twice shorter than cymbium, with three apophyses; retrolateral tibial apophysis (RTA) about 50% of tibia length, heavily sclerotised and with a fingerlike apex; prolateral tibial apophysis (PTA) distinctly elevated and relatively short, about 30% of tibia length, coniform in prolateral view and digitiform in ventral view; dorsal tibial apophysis (DTA) thin and stalk-shaped, about as long as RTA; cymbial spur (CS) beak-shaped, twice shorter than tibia; cymbial fold (CF) strongly developed and well visible in ventral and retrolateral view, for approximately 2/3 the length of cymbium; tip of cymbium long, about 1/3 of cymbium length. Tegulum 1.3 longer than wide, membranous and semitransparent except its margin in ventral view; tegular apophysis (TA) short and thin, spiculate; embolus (E) starts on the retrolateral flank (approximately 3 o’clock of tegulum), surrounds the base and ends at conductor (C) apex, its tip filiform and curved behind conductor; conductor large and membranous.

***Female***. Unknown.

##### Comments.

According to the World Spider Catalogue 2018, a total of 11 *Cheiracanthium* species are known from females only in China: *C. approximatum* O. P.-Cambridge, 1885, *C. escaladae* Barrion et al., 2013, *C. fujianense* Gong, 1983, *C. hypocyrtum* Zhang & Zhu, 1993, *C. liuyangense* Xie et al., 1996, *C. olliforme* Zhang & Zhu, 1993, *C. potanini* Schenkel, 1963, *C. rupicola* (Thorell, 1897), *C. solidum* Zhang et al., 1993, *C. sphaericum* Zhang et al., 1993, *C. longtailen* Xu, 1993. Among them, *C. escaladae* is supposedly a *Clubiona* species based on epigyne morphology, while *C. potanini* is supposededly doubtful because of the poor original illustrations and description. The other nine can be considered tentatively as valid *Cheiracanthium* species. However, none of them could be matched with *C. echinulatum* sp. n. due to their different habitus (abdomen without distinct colour pattern in *C. echinulatum* sp. n., but with a median heart-shaped mark in *C. approximatum*, *C. fujianense* and *C. rupicola*, with two pairs of muscular depressions in *C. hypocyrtum, C. liuyangense* and *C. sphaericum*) and different number of cheliceral teeth (chelicerae with 3 promarginal and 3 retromarginal teeth in *C. echinulatum* sp. n., but with 3 promarginal and 2 retromarginal teeth in *C. hypocyrtum*, *C. olliforme* and *C. sphaericum*, with 3 promarginal and 1 retromarginal teeth in *C. liuyangense*, with 2 promarginal and 1 retromarginal teeth in *C. solidum*, with 2 promarginal and 3 retromarginal teeth in *C. longtailen*).

##### Distribution.

Presently known only from the type locality, Jinggang Mountains, Jiangxi, China (Fig. 5).

#### Cheiracanthium taiwanicum

Taxon classificationAnimaliaAraneaeEutichuridae

Chen, Huang, Chen & Wang, 2006

Figs 3 , 5

Cheiracanthium taiwanicum Chen et al., 2006: 10, fig. 1A–E; Chen and Huang 2012: 25, fig. 7A–G, pl. 2C–D, 3A–B.

##### Examined material.

1♂ and 1♀, China, Jiangxi Province, Jinggang Mountains Nature Reserve, Hexiliong village (680 m; 26°31'51.54"N, 114°8'46.02"E), 30 April 2011, Hao Yu leg.

##### Description.

**Figures 3.**
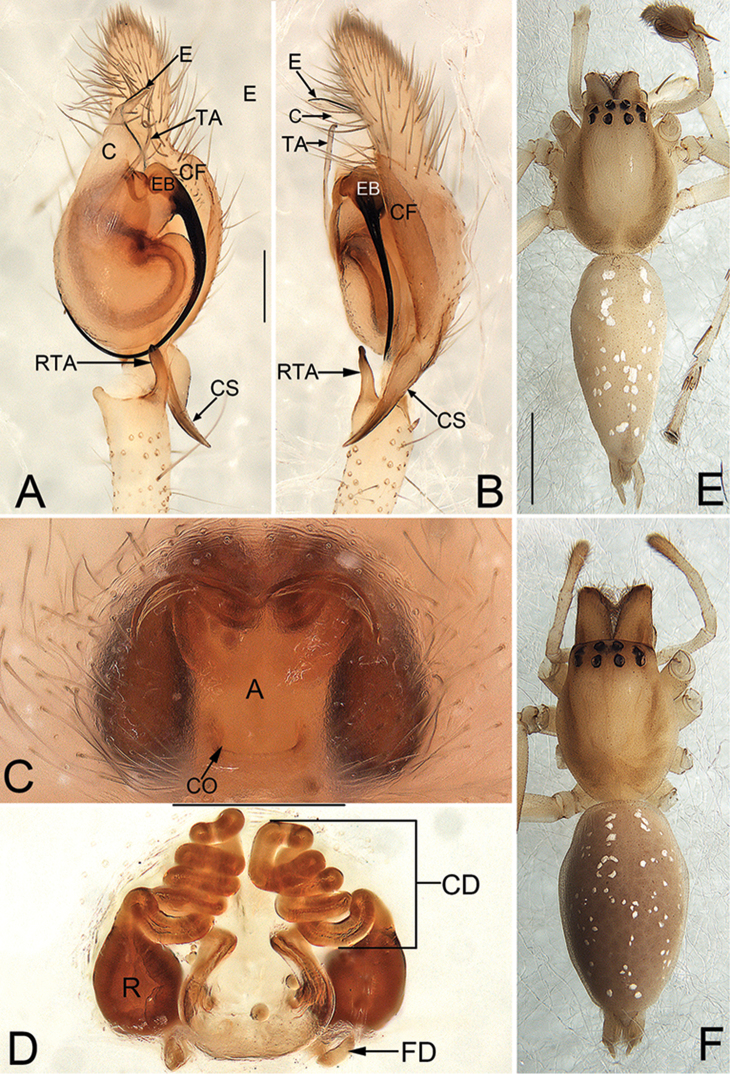
*Cheiracanthium taiwanicum* Chen, Huang, Chen & Wang, 2006, male and female from Jinggang Mountains, Jiangxi, China. **A** left male palp, ventral view **B** same, retrolateral view **C** epigyne, ventral view **D** vulva, dorsal view **E** male habitus, dorsal view **F** female habitus, dorsal view. Scale bars: 0.2 mm (**A–B, C–D**); 1 mm (**E–F**).

*Male* and *female* (Fig. 3). For details see Chen and Huang (2012).

##### Distribution.

Jinggang Mountains in Jiangxi and Nantou County in Taiwan, China.

#### Cheiracanthium zhejiangense

Taxon classificationAnimaliaAraneaeEutichuridae

Hu & Song, 1982

Figs 4 , 5

Cheiracanthium zhejiangensis Hu & Song, 1982: 56, fig. 4A–D.
Cheiracanthium zhejiangense Paik 1990: 9, fig. 26–38.

##### Remarks.

See the World Spider Catalogue for the full list of references.

##### Examined material.

1♂ and 2♀, China, Jiangxi Province, Jinggang Mountains Nature Reserve, Xiangzhou village (380 m; 26°35'30.23"N, 114°15'59.93"E), 26 April 2011, Hao Yu leg.

##### Description.

**Figures 4.**
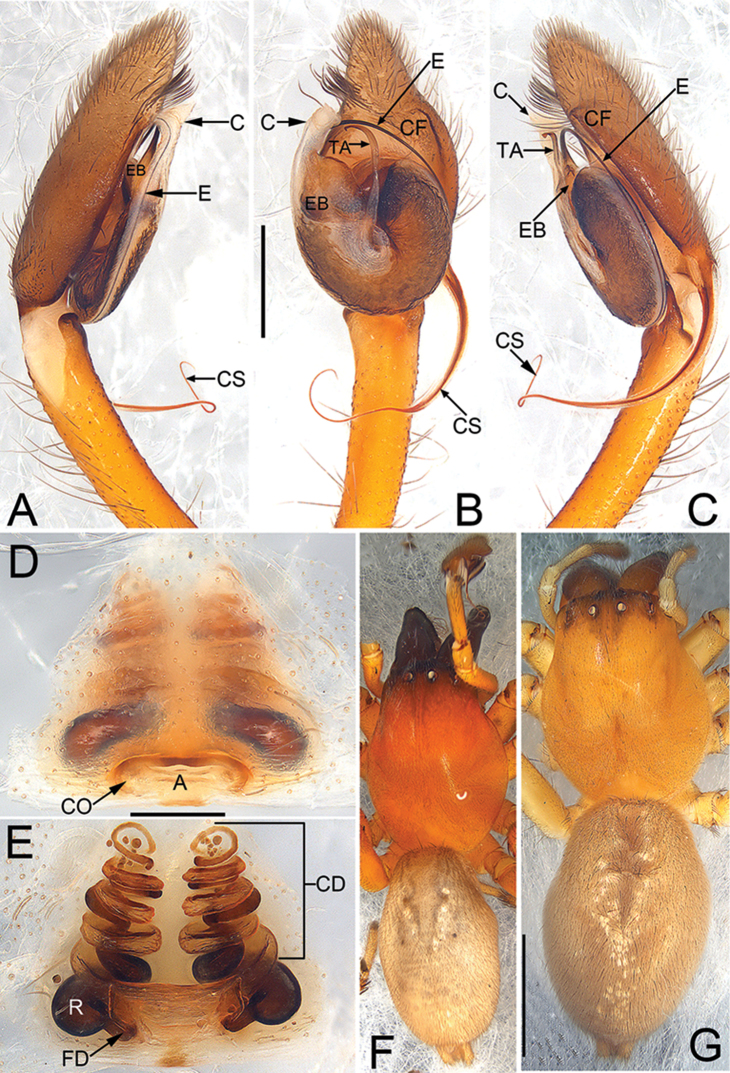
*Cheiracanthium zhejiangense* Hu & Song, 1982, male and female from Jinggang Mountains, Jiangxi, China. **A** left male palp, prolateral view **B** same, ventral view **C** same, retrolateral view **D** epigyne, ventral view **E** vulva, dorsal view **F** male habitus, dorsal view **G** female habitus, dorsal view. Scale bars: 0.5 mm (**A–C**); 0.2 mm (**D–E**); 2 mm (**F–G**).

*Male* and *female* (Fig. 4). Description of habitus, see Paik (1990). Since previous descriptions are rather brief, redescription of genitalia is provided as below.

*Palp* (Fig. 4A–C). Tibia about as long as cymbium, with only retrolateral apophysis; apophysis about 20% of tibia length, with a sharp apex and hiding behind tegulum; cymbial spur slightly shorter than tibia, tapering off into a thread and terminally coiled; cymbial fold distinct, for approximately 2/3 the length of cymbium; tip of cymbium about 1/3 of cymbium length. Tegulum egg-shaped, 1.2 longer than wide; tegular apophysis long, more than 4/5 of tegulim length, thin hook-shaped; embolus originates at about 10 o’clock position, terminating at approximately 11 o’clock position, it’s tip covered by conductor; conductor large, membranous.

*Epigyne* (Fig. 4D–E). Apron-like atrium distinct, about four times wider than long; receptacles are faintly visible through epigynal plate in ventral view; two copulatory openings located at lateral borders of atrium; the transparent copulatory ducts running spirally (length of spira about 2.6 times longer than receptacles), forming 8 entwined loops (including 4 ascending coils and 4 descending coils); receptacle long and tubular, separated by two diameters.

##### Distribution.

China (Guizhou, Hunan, Jiangxi, Zhejiang) and Korea.

**Figure 5.**
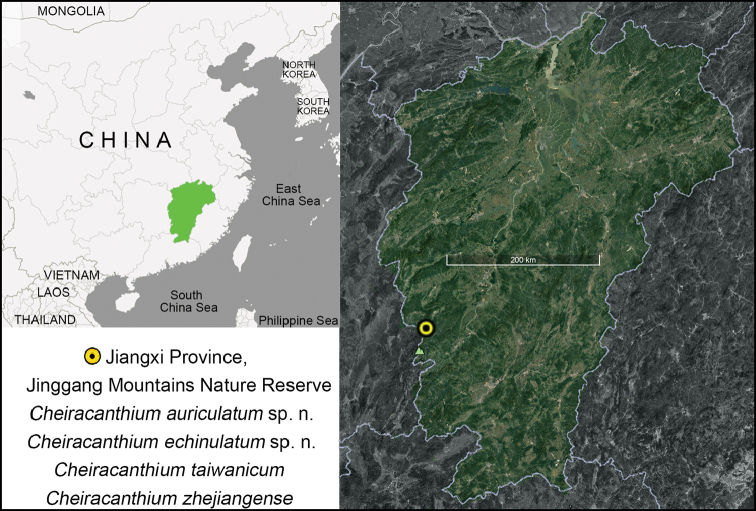
Locality of Jinggang Mountains in China.

## Supplementary Material

XML Treatment for Cheiracanthium auriculatum

XML Treatment for Cheiracanthium echinulatum

XML Treatment for Cheiracanthium taiwanicum

XML Treatment for Cheiracanthium zhejiangense
